# 3D Organotypic Spinal Cultures: Exploring Neuron and Neuroglia Responses Upon Prolonged Exposure to Graphene Oxide

**DOI:** 10.3389/fnsys.2019.00001

**Published:** 2019-01-24

**Authors:** Mattia Musto, Rossana Rauti, Artur Filipe Rodrigues, Elena Bonechi, Clara Ballerini, Kostas Kostarelos, Laura Ballerini

**Affiliations:** ^1^Neuron Physiology and Technology Lab, International School for Advanced Studies (SISSA), Trieste, Italy; ^2^Nanomedicine Lab, Faculty of Biology, Medicine & Health and National Graphene Institute, University of Manchester, Manchester, United Kingdom; ^3^Department NEUROFARBA, University of Florence, Florence, Italy; ^4^Laboratory of Neuroimmunology, Dipartimento di Medicina Sperimentale e Clinica, University of Firenze, Firenze, Italy

**Keywords:** graphene oxide, organotypic cultures, patch-clamp, microglia, microvesicles

## Abstract

Graphene-based nanomaterials are increasingly engineered as components of biosensors, interfaces or drug delivery platforms in neuro-repair strategies. In these developments, the mostly used derivative of graphene is graphene oxide (GO). To tailor the safe development of GO nanosheets, we need to model *in vitro* tissue responses, and in particular the reactivity of microglia, a sub-population of neuroglia that acts as the first active immune response, when challenged by GO. Here, we investigated central nervous system (CNS) tissue reactivity upon long-term exposure to GO nanosheets in 3D culture models. We used the mouse organotypic spinal cord cultures, ideally suited for studying long-term interference with cues delivered at controlled times and concentrations. In cultured spinal segments, the normal presence, distribution and maturation of anatomically distinct classes of neurons and resident neuroglial cells are preserved. Organotypic explants were developed for 2 weeks embedded in fibrin glue alone or presenting GO nanosheets at 10, 25 and 50 μg/mL. We addressed the impact of such treatments on premotor synaptic activity monitored by patch clamp recordings of ventral interneurons. We investigated by immunofluorescence and confocal microscopy the accompanying glial responses to GO exposure, focusing on resident microglia, tested in organotypic spinal slices and in isolated neuroglia cultures. Our results suggest that microglia reactivity to accumulation of GO flakes, maybe due to active phagocytosis, may trim down synaptic activity, although in the absence of an effective activation of inflammatory response and in the absence of neuronal cell death.

## Introduction

Graphene is a monolayer sheet of carbon atoms, tightly bound in a hexagonal honeycomb lattice. More specifically, graphene is an allotrope of carbon in the form of a two-dimensional film of sp^2^ hybridized carbon atoms (Sanchez et al., 2012; Kostarelos and Novoselov, 2014), characterized by high mechanical strength and electrical conductivity, combined with optical transparency. In neurobiology, graphene has been used in surface engineering of regenerative scaffolds to control the neuro-induction of stem cells (Wang et al., 2012), and in that of neurological interfaces to improve electrode performance (Wang et al., 2011; Li et al., 2013; Mao et al., 2013; Kostarelos and Novoselov, 2014).

Graphene oxide (GO) is the most common derivative of graphene. Recently, GO materials have been successfully designed for drug delivery applications (Yang et al., 2008, 2011; Liu et al., 2013; Baldrighi et al., 2016; Rauti et al., 2018). However, their potential persistence in biological tissues requires investigating their safety. We have previously (Rauti et al., 2016) reported the ability of small GO (<200 nm; s-GO) nanosheets to reduce synaptic activity at glutamatergic synapses without affecting cultured hippocampal neurons survival. To date, only few studies addressed the interaction between s-GO nanosheets and synapses (Bramini et al., 2016; Rauti et al., 2016), while there are scarcely any data on the interactions between neural circuit function, s-GO tissue accumulation and inflammation. Before any further exploitation of s-GO in synaptic targeting, a detailed analysis of tissue responses to s-GO exposure is needed.

Mechanistic studies of the interplay between s-GO, the activation of microglia and synaptic function, may require *in vitro* models to interrogate central nervous system (CNS) responses at cellular resolution. Organotypic slices are explant cultures that preserve key, structural elements of the tissue of origin (Hailer et al., 1996; Fischer et al., 1998; Tscherter et al., 2001; Schermer and Humpel, 2002; Avossa et al., 2003, 2006; Furlan et al., 2007; Medelin et al., 2016) allowing detailed studies of cellular and subcellular responses, such as inflammatory reactivity and synaptic efficacy (Medelin et al., 2018), upon chronic treatments, including the exposure to exogenous factors. In the CNS, the immune response is mediated by resident macrophages called microglia that are approximately 12% of the total CNS cells originating from myeloid cells. This subpopulation of brain cells can switch between two different phenotypes: a ramified phenotype, typical of the resting state, during which they “monitor” the surrounding environment (Davalos et al., 2005; Nimmerjahn et al., 2005; Cherry et al., 2014) and an ameboid phenotype, which is induced by antigen-mediated stimulation. When activated, microglia rapidly changes its surface receptor expression and the production of molecules involved in the immune response, like cytokines and chemokines (Fetler and Amigorena, 2005; Nimmerjahn et al., 2005). Activated microglia may represent an active player in neuron damage (Block et al., 2007).

We used mouse spinal organotypic cultures to mimic a chronic accumulation of s-GO in the spinal cord tissue. The s-GO nanosheets were delivered to the spinal tissue upon dilution in the chicken plasma (fibrin glue) used to embed the explants for culturing, thus allowing s-GO to rapidly adsorb proteins (Bertrand et al., 2017), to mimic how nanosheets behave in a complex biological milieu. We patch-clamped ventral interneurons to monitor synaptic transmission. Contextually, using confocal microscopy we explored the effects of s-GO on innate immunity, in both organotypic slices and primary isolated microglial cultures. We conclude that chronic accumulation of s-GO, due to localization of high doses of the material, significantly affected synaptic activity and the microglia cell population. Our experiments in isolated microglial cells in culture support the direct response of these cells to s-GO in these experimental conditions, however in organotypic cultures we did not detect strong indicators of a switch toward the pro-inflammatory phenotype.

## Materials and Methods

### Preparation of s-GO

Synthesis and characterization of s-GO used in the present study were fully described in a separate article (Rodrigues et al., 2018). Briefly, 0.8 g of graphite flakes (Graflake 9580, Nacional Grafite Ltda., Brazil) were oxidized after mixing with 0.4 g of sodium nitrate (Merck-Sigma, UK) and 18.4 mL of sulfuric acid 99.999% (Merck-Sigma, UK), followed by 2.4 g of potassium permanganate (Merck-Sigma, UK), according to the modified Hummers method, which was described in our previous work (Ali-Boucetta et al., 2013; Rauti et al., 2016). After mixing for 30 min, 37.5 mL of water for injections (Fresenius Kabi Ltd., UK) were added dropwise to ensure the safety of the exothermic reaction taking place. The reaction temperature was further increased to 98°C, and maintained for another 30 min. The reaction was terminated after adding 112.5 mL of water for injections, followed by the dropwise addition of 12.5 mL of hydrogen peroxide 30% (Merck-Sigma, UK). The resulting mixture was purified by several rounds of centrifugation at 9,000 rpm for 20 min, until the supernatant reached a neutral pH and a viscous orange/brown layer of pure GO appeared on top of the pelleted oxidation byproducts (Jasim et al., 2016; Rauti et al., 2016). GO was exfoliated using warm water for injections and purified by centrifugation at 4,000 rpm for 20 min to remove any residual graphitic impurities. The obtained GO dispersion was aliquoted to depyrogenated glass vials, which were exposed to a water bath sonicator (VWR, UK) operating at 80 W (45 kHz) for 5 min. The sonicated dispersion was then centrifuged at 13,000 rpm for 5 min, and the supernatant was collected yielding s-GO nanosheets. All procedures were conducted under endotoxin-free conditions, which were attained by setting the reaction under a laminar flow hood, using depyrogenated glassware and nonpyrogenic plastic containers (Mukherjee et al., 2016).

Atomic force microscopy (AFM) images of s-GO were acquired using a Multimode 8 microscope (Bruker, UK), operating in tapping mode using OTESPA tips (Bruker, UK). Twenty microliter of s-GO, diluted to a concentration of 100 μg/mL, were deposited on top of a freshly prepared mica surface (Agar Scientific, UK) coated with poly-L-lysine (Sigma-Aldrich, UK), and dried overnight at 37°C prior to analysis. Full physicochemical characterization of s-GO is summarized in Supplementary Figure S1.

### Preparation of Spinal Tissue Slices and Primary Glial Cultures

Organotypic cultures were obtained from spinal cords isolated from E12 embryonic mouse (C57Bl), as previously described (Avossa et al., 2003; Furlan et al., 2005, 2007; Usmani et al., 2016). Briefly, pregnant mice were sacrificed by CO_2_ overdose and decapitation and fetuses delivered by cesarean section. Isolated fetuses were decapitated and their backs were isolated from low thoracic and high lumbar regions and transversely sliced (275 μm) with a tissue chopper. Cultures were fixed on a glass coverslip (Kindler, EU) with fibrin glue, i.e., reconstituted chicken plasma (Rockland) clotted with thrombin (Merk). In graphene-treated cultures, s-GO (Rauti et al., 2016) nanosheets were embedded in the fibrin glue at 10, 25 and 50 μg/mL final concentration. The distribution of s-GO within this matrix was detected *via* confocal microscopy under reflection mode (see below; and Patskovsky et al., 2015). Supplementary Figure S2 shows the dispersion of s-GO at the different concentrations. Experiments were performed on control and s-GO treated cultures after 2 and 3 weeks *in vitro*.

All experiments were performed in accordance with the EU guidelines (Directive 2010/63/EU) and Italian law (decree 26/14) and were approved by the local authority veterinary service and by our institution (SISSA-ISAS) ethical committee. All efforts were made to minimize animal suffering and to reduce the number of animal used. Animal use was approved by the Italian Ministry of Health, in agreement with the EU Recommendation 2007/526/EC.

Primary brain glial cultures were obtained from P2 to P3 rats (Wistar) cortices, as previously described (Calegari et al., 1999; Rauti et al., 2016). Dissociated cells were plated into plastic 75 cm^2^ flasks, incubated (37°C; 5% CO_2_) in culture medium consisting of DMEM (Invitrogen, Carlsbad, CA, USA), supplemented with 10% fetal bovine serum (FBS), 100 IU/mL penicillin, and 10 mg/mL streptomycin.

Confluent mixed glial cultures from days *in vitro* (DIV) 21 to DIV 25 were treated with a trypsin solution (0.25% trypsin, 1 mM EDTA in HBSS) diluted 1:4 in PBS for 30 min at 37°C and 5% CO_2_. The medium was then collected and diluted 1:4 in DMEM supplemented with 10% FBS and centrifuged for 5 min at 200× *g*. The pellet was then re-suspended in DMEM supplemented with 10% FBS and mixed glial cultures conditioned medium (50:50) and plated on poly-L-lysine-coated glass coverslips. Twenty-four hours after trypsinization half of the cultures were incubated with s-GO at a concentration of 10 μg/mL suspended in the culture medium for 1 or 5 days.

### Electrophysiological Recordings

For patch-clamp recordings (whole-cell, voltage clamp mode), a coverslip with the spinal culture was positioned in a recording chamber, mounted on an inverted microscope (Eclipse TE-200, Nikon, Japan) and superfused with control physiological saline solution containing (in mM): 152 NaCl, 4 KCl, 1 MgCl_2_, 2 CaCl_2_, 10 HEPES and 10 Glucose. The pH was adjusted to 7.4 with NaOH (osmolarity 305 mosmol L^−1^). Cells were patched with glass pipettes (4–7 MΩ) filled with a solution of the following composition (in mM): 120 Kgluconate, 20 KCl, 10 HEPES, 10 EGTA, 2 MgCl_2_ and Na_2_ATP. The pH was adjusted to 7.3 with KOH (295 mosmol L^−1^). All electrophysiological recordings were performed at room temperature (RT; 20–22°C) and the spontaneous synaptic activity was recorded by clamping the membrane voltage at −56 mV (not corrected for liquid junction potential, which was −14 mV). Recordings were performed from ventrally located spinal interneurons identified on the basis of previously reported criteria (Ballerini and Galante, 1998; Ballerini et al., 1999; Galante et al., 2000). We detected no differences between controls (*n* = 45) and s-GO (*n* = 39) neurons in cell membrane capacitance (70 ± 8 pF controls, 68 ± 6 pF s-GO) and membrane input resistance (250 ± 28 MΩ controls, 242 ± 20 MΩ s-GO). Spontaneous activity was also recorded in the presence of 6-cyano-7-nitroquinoxaline-2,3-dione (CNQX, 10 μM), bicuculline (20 μM) and strychnine (10 μM) to pharmacologically discriminate between glutamatergic and GABAergic postsynaptic currents (PSCs), respectively. To detect miniature PSCs (mPSCs), tetrodotoxin (TTX, 1 μM; Latoxan, Valence, France) was added. All reagents were purchased from Sigma-Aldrich, if not otherwise indicated. Data were collected by Multiclamp 700B patch amplifier (Axon CNS, Molecular Devices, San Jose, CA, USA) and digitized at 10 kHz with the pClamp 10.2 software (Molecular Devices LLC, San Jose, CA, USA). All recorded events were analyzed offline with the AxoGraph 1.4.4 (Axon Instrument) event detection software (Axon CNS, Molecular Devices, San Jose, CA, USA).

### Immunofluorescence Labeling of Spinal Cord Slices

Organotypic cultures were fixed by 4% formaldehyde (prepared from fresh paraformaldehyde; Sigma) in PBS for 1 h at RT and then washed in PBS. Free aldehyde groups were quenched in 0.1 M glycine in PBS for 5 min. The samples were blocked and permeabilized in 3% FBS, 3% BSA and 0.3% Triton-X 100 in PBS for 1 h at RT. Samples were incubated with primary antibodies (mouse anti-neurofilament H Smi 32, Biolegend, 1:250 dilution; mouse monoclonal anti-GFAP, Invitrogen, 1:500 dilution; rabbit monoclonal anti-caspase 3, Euroclone, 1:200 dilution; rabbit polyclonal anti-β-tubulin III, Sigma-Aldrich, 1:250 dilution; rabbit anti Iba1, Wako, 1:250 dilution) diluted in PBS with 5% FBS at 4°C, overnight. Samples were then incubated in secondary antibodies (Alexa 488 goat anti-mouse, Invitrogen, 1:500 dilution; Alexa 594 goat anti-rabbit, Invitrogen, 1:500 dilution), and DAPI (Invitrogen, dilution 1:200) to stain the nuclei, for 2 h at RT and finally mounted on 1 mm glass coverslips using Vectashield hardset mounting medium (Vector Laboratories). Images were acquired using a Nikon C2 Confocal, equipped with Ar/Kr, He/Ne and UV lasers. Images were acquired with a 40× (1.4 NA) or 60× (1.5 NA) oil-objective (using oil mounting medium, 1.515 refractive index). Confocal sections were acquired every 500 nm and the total Z-stack thickness (50 μm) was set such that all emitted fluorescence was collected from the sample. Regions of interest were confined to the ventral part of slice. Offline analysis was performed using the open source image-processing package Fiji (Schindelin et al., 2012) and Volocity software (Volocity 3D image analysis software, PerkinElmer, Waltham, MA, USA).

### Immunofluorescence Labeling of Neuroglia Primary Cultures

Primary glial and microglial cultures were fixed by 4% formaldehyde (prepared from fresh paraformaldehyde) in PBS for 20 min at RT and then washed in PBS. Free aldehyde groups were quenched in 0.1 M glycine in PBS for 5 min. The samples were blocked and permeabilized in 5% FBS, 0.3% Triton-X 100 in PBS for 30 min at RT. Samples were incubated with primary antibodies (mouse monoclonal anti-GFAP, Invitrogen, 1:500 dilution; rabbit anti Iba1, Wako, 1:250 dilution; mouse monoclonal anti-bromodeoxyuridine (Brdu), Thermo Fisher, 1:200 dilution) diluted in PBS with 5% FBS at 4°C, overnight. Samples were then incubated in secondary antibodies (Alexa 488 goat anti-mouse, Invitrogen, 1:500 dilution; Alexa 594 goat anti-rabbit, Invitrogen, 1:500 dilution), and DAPI (Invitrogen, dilution 1:200) to stain the nuclei, for 45 min at RT and finally mounted on 1 mm thick glass coverslips using Vectashield mounting medium (Vector Laboratories). Cells densities were quantified at 20× (0.5 NA) magnification using a DM6000 Leica microscope (Leica Microsystems GmbH, Wetzlar, Germany). In order to investigate the internalization of s-GO in microglial cells, we used the reflection mode property during the confocal acquisition. Images were acquired using a Nikon C2 Confocal, equipped with Ar/Kr, He/Ne and UV lasers. Images were acquired with a 40× (1.4 NA) oil-objective (using oil mounting medium, 1.515 refractive index). Confocal sections were acquired every 200 nm and the total Z-stack thickness 20 μm.

### Bromodeoxyuridine (BrdU) Incorporation

Microglial primary cultures were incubated with BrdU (Thermo Fisher, Waltham, MA, USA) diluted in the culture medium at a final concentration of 10 μM for 24 h. Cells were then washed with PBS and fixed by 4% formaldehyde (prepared from fresh paraformaldehyde) for 20 min at RT and then washed with PBS (three times, 2 min each). Free aldehyde groups were quenched in 0.1 M glycine in PBS for 5 min. Cells were then incubated with HCl 1 M for 1 min on ice and with HCl 2 M for 15 min at 37°C. Acid was then neutralized with boric acid 0.1 M for 10 min at RT. The samples were then blocked and permeabilized in 3% FBS and 0.3% Triton-X 100 in PBS for 1 h at RT. Samples were incubated with primary antibodies at 4°C overnight (mouse monoclonal anti-BrdU, 1:200 dilution; rabbit anti-Iba1, 1:200 dilution). Samples were then incubated in secondary antibodies (Alexa 488 goat anti-mouse, Invitrogen, 1:500 dilution; Alexa 594 goat anti-rabbit, Invitrogen, 1:500 dilution), and DAPI (Invitrogen, dilution 1:200) to stain the nuclei, for 45 min at RT and mounted on 1 mm thick glass coverslips using Vectashield mounting medium (Vector Laboratories).

### Microvesicle Isolation

Microvesicles (MVs) shedding and detection by western blotting were performed as previously described (Rauti et al., 2016). MVs release was induced in 21 DIV microglial cells by the stimulation with benzoyl-ATP (bzATP; 100 μM) in saline solution with the following composition: 125 mM NaCl, 5 mM KCl, 1.2 mM MgSO_4_, 1.2 mM KH_2_PO_4_, 2 mM CaCl_2_, 6 mM D-glucose, and 25 mM HEPES/NaOH (pH adjusted to 7.4), for 30 min at 37°C and 5% CO_2_. MVs were then pelleted by centrifugation (Bianco et al., 2009). Negative controls were incubated with saline solution without the presence of bzATP. MVs were re-suspended in lysis buffer (50 mM Tris-HCl, pH 8.0, 150 mM NaCl, 1% NP40, 0.1% SDS), sonicated 3 × 10 s, and then boiled at 95°C for 5 min. Samples were run on a 10% polyacrylamide gel and were blotted onto nitrocellulose filters (Millipore, Italy). Filters were then blocked in PBS-Tween-20 (0.1%) plus 5% nonfat dry milk and incubated with the primary antibody antiflotillin-1 (dilution 1:1,000) for 16 h at 4°C. Specific MV marker flotillin-1 (del Conde et al., 2005; Al-Nedawi et al., 2008) was detected with mouse monoclonal antiflotillin-1 (dilution 1:1,000). After three washes with PBS-Tween, filters were incubated with peroxidase-conjugated anti-mouse secondary antibody (dilution 1:1,000). Optical density of immunolabeled ECL-exposed protein bands was measured with UVI-1D software.

### Microglial Morphological Analysis

For morphological analysis cells were fixed and immunostained for Iba1 and DAPI for nuclei, as described above and images were acquired with a 40× oil objective. The quantitative analysis of cell morphology was performed with the particle analysis feature in Fiji (1.51v) to automatically measure the area, perimeter and Feret’s maximum diameter. In particular, Feret’s diameter is described as the greatest distance between any two points along cell perimeter and is considered as an index of cell length (Kurpius et al., 2006; Caldeira et al., 2014; Torres-Platas et al., 2014; Zanier et al., 2015; Mitchell et al., 2018). A more ramified cell has a higher value for this parameter, while a more ameboid shape is described by a lower value. We also evaluated the transformation index (Fujita et al., 1996; Caldeira et al., 2014) which is calculated as [perimeter of cell (μm)]^2^/4π [cell area (μm^2^)] and describes the degree of cellular ramification. Cells with long processes and small cell body display larger values of the transformation index, which depends on the cell shape regardless the cell size.

### Measurement of Cytokines and Chemokines

In a small set of experiments an inflammatory response was induced (Hanisch, 2002), by incubating organotypic slices, for 6 h, with a cocktail of the following mouse recombinant cytokines: TNF-α (R&D Systems, #210-TA/CF), IL-1β (R&D Systems, #M15330), GM-CSF (R&D Systems, #P04141), 10 ng/mL each, or with lipopolysaccharide (LPS, 1 ng/mL). Inflammatory reaction may be detected by cytokine and chemokine production. A panel of 12 out of cytokines or chemokines was measured in organotypic culture supernatants after 2 weeks culturing, by Luminex based technology, using a customized Procarta plex Immunoassay kit (Invitrogen, Carlsbad, CA, USA), following the manufacturer’s protocol. The following soluble factors were simultaneously measured in 50 μl of supernatant: IL4, IL6, IL10, IL17, IL21, BAFF, IFNγ, TNFα, CXCL1, CXCL2, CXCL10, MCP1.

### Statistical Analysis

The results are presented as the mean ± SD, if not otherwise indicated. Statistically significant difference between two data sets was assessed by t-statistic, in particular by Student’s *t*-test (after checking variances homogeneity by Leven’s test) for parametric data and by Mann-Whitney’s test for non-parametric ones (Statistica 6.0—StatSoft, Italy). Differences among multiple groups were evaluated by F-statistic with two-way ANOVA, followed by the Holm-Sidak test for multiple comparison (Sigmaplot 12.0—Systat Software).

A statistically significant difference between two data sets was assessed and *P* < 0.05 was considered statistically significant.

In box-plots, the thick horizontal bar indicates the median value, the cross indicates the mean value, the boxed area extends from the 25th to 75th percentiles while whiskers from the 5th to the 95th percentiles.

## Results

### Long-Term Exposure to High Doses of s-GO Impaired Network Activity in Spinal-Cord Organotypic Slices

**Figure 1:**
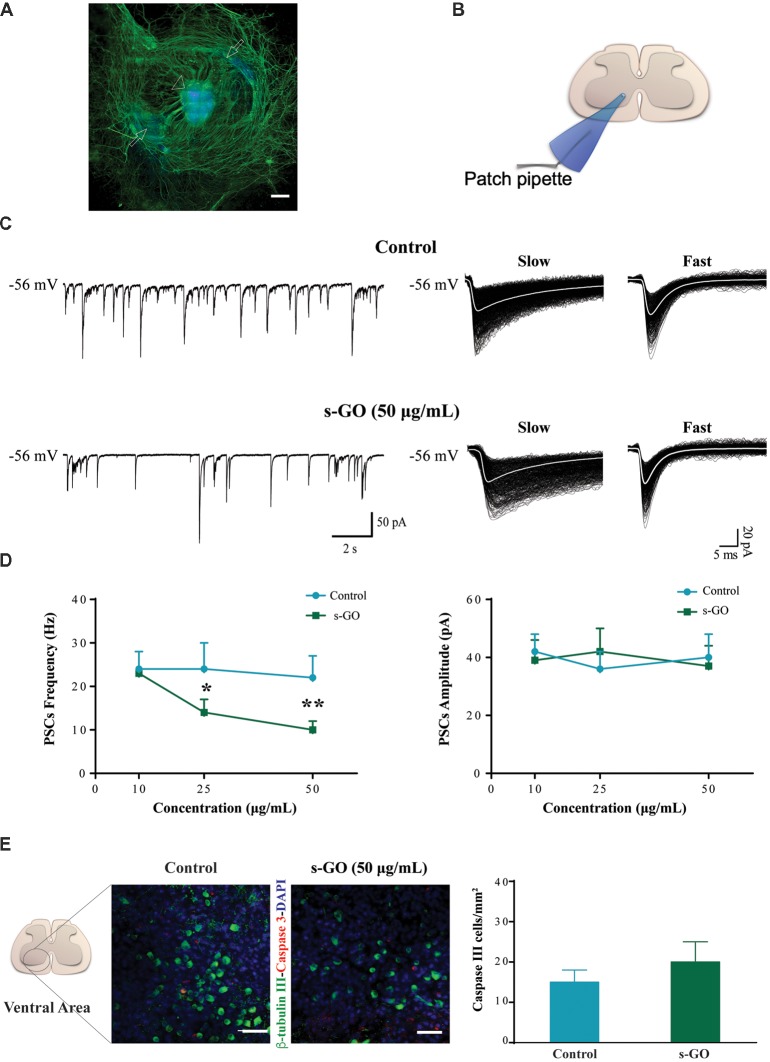
s-graphene oxide (s-GO) reduced basal synaptic activity in a dose-response fashion without inducing cell death in organotypic ventral horns. In **(A)**, low magnification confocal micrograph of a spinal cord slice culture (14 days *in vitro*, DIV) immune-labeled for neurofilament H (SMI-32; in green) and nuclei (DAPI; in blue). Scale bar 500 μm. The arrow head indicates the ventral fissure, localizing the ventral horns, while the arrows the co-cultured dorsal root ganglias (DRGs). In **(B)**, sketch of the experimental setting for ventral interneuron single cell recordings (modified with permission from Usmani et al., 2016). In **(C)**, tracings represent spontaneous synaptic activity recorded from ventral interneurons in Control (top) and s-GO treated slices (bottom; 50 μg/mL). On the right panel, isolated fast and slow postsynaptic currents (PSCs) are shown superimposed (electronic average trace superimposed in white) for the control (top) and s-GO treated (bottom) recordings (same cells as above). In **(D)**, the plots summarize the average PSCs frequency (left) and amplitude (right) values; note the reduction in PSC frequency upon s-GO treatments (25 and 50 μg/mL; *P < 0.05 and **P < 0.01). In **(E)**, confocal micrographs visualize caspase-3 positive cells (in red) in the ventral horns, counter-stained for β-tubulin III (in green, to visualize neurons) in Control (left) and s-GO (50 μg/mL; right) treated slices. Nuclei are visualized by DAPI (blue). Scale bar: 50 μm. The column plot summarizes the density of caspase and β-tubulin double positive cells; note the absence of statistical significant differences between the two conditions.

We first explored the long-term (2 weeks) exposure of neural tissue to s-GO in 3D tissue cultures. s-GO was delivered to the neural tissue *via* the fibrin glue, the thick matrix obtained by chicken plasma and thrombin in which slices are embedded (see “Materials and Methods” section), that represents the explant growth environment. Figure 1A shows a reconstruction at low confocal magnification of a spinal cord slice after 14 days of growth, labeled for neurofilament H (Smi-32; in green) and for the nuclei (DAPI, in blue). The entire area of tissue growth is visualized, and it includes the spinal slice, at the center, and the outgrowing area comprising the co-cultured dorsal root ganglia (DRG) and the typical, dense mesh of Smi-32^+^ neurites in the surrounding outgrowth belt (Fabbro et al., 2012).

Spinal organotypic slices upon 2 weeks of culturing exhibit an intense spontaneous synaptic activity (Streit, 1993; Ballerini and Galante, 1998; Furlan et al., 2007). We patch clamped (sketched in Figure 1B) visually identified ventral interneurons (at holding potential, V_h_ of −56 mV) in control cultures (*n* = 45) and in s-GO treated ones (*n* = 52), and we recorded spontaneous, basal PSCs. Figure 1C shows representative tracings in control (top) and after exposure to the highest dose (50 μg/mL) of s-GO (bottom). In all culture groups, PSCs appeared as heterogeneous inward currents of variable amplitudes, characterized by different kinetic properties (fast decaying events, with decay time constant (τ) of 6 ± 2 ms and slow decaying events with τ 22 ± 6, *n* = 15, see sample in Figure 1C, right panel; Medelin et al., 2018).

The chronic (2 weeks) exposure to low (10 μg/mL) doses of s-GO did not affect PSCs amplitude and frequency values (39 ± 7 pA and 23 ± 5 Hz, *n* = 13) when compared to control ones (42 ± 6 pA and 24 ± 4 Hz, *n* = 15; plots in Figure 1D). Conversely, higher s-GO doses significantly (25 and 50 μg/mL, *P* < 0.05 and *P* < 0.01, respectively; two-way ANOVA) reduced PSCs frequency (from 24 ± 6 Hz in control to 14 ± 3 Hz, in s-GO 25 μg/mL, *n* = 15 and 13; from control 22 ± 8 Hz to 10 ± 2 Hz in s-GO 50 μg/mL, *n* = 15 and 13; plot in Figure 1D, left). Upon s-GO treatments, PSCs decay kinetics (fast decaying, τ 5 ± 2 ms and slow decaying τ 26 ± 3, *n* = 13 at 50 μg/mL, see sample Figure 1C bottom row, right) and amplitudes were not altered by these treatments (Figure 1D, right). In 25 and 50 μg/mL s-GO treated cultures we investigated the amount of neuronal apoptosis in respect to aged-matched controls by measuring the expression of active caspase-3 (Cohen, 1997). Active caspase-3 positive cells were quantified in the ventral spinal horns (Figure 1E, in red). We detected a comparative amount of apoptotic cells in all conditions (in control: 15 ± 3 Caspase-3 positive cells/mm^2^ namely 3.7% of the total amount of cells, *n* = 10 visual fields and in s-GO 50 μg/mL; 20 ± 3 Caspase-3 positive cells/mm^2^, 4.3% of the cells, *n* = 10 visual fields; plot in Figure 1E). Thus, s-GO only when delivered at higher concentrations altered synapse function, without increasing neuronal cell death.

**Figure 2:**
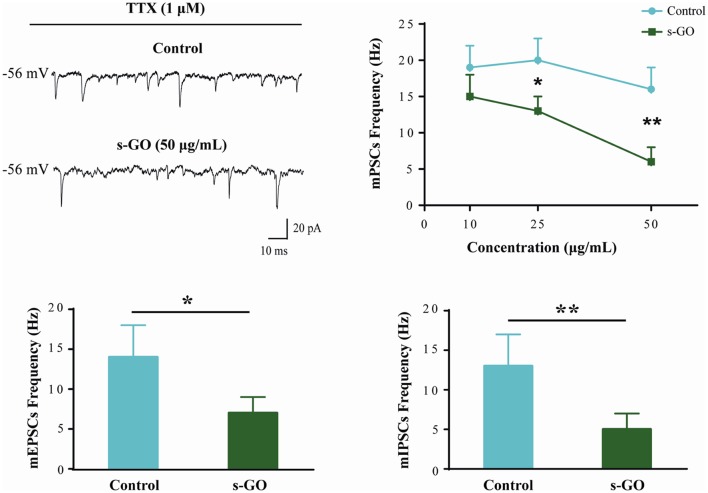
Miniature PSCs (mPSCs) are down regulated by s-GO in organotypic ventral horns. Sample tracings are mPSCs recorded in Control and s-GO (50 μg/mL) treated cultures (top left panel). Top right: plot reporting mPSCs frequency values in control and in the three different s-GO concentrations tested. Note that s-GO treatments (25 and 50 μg/mL) significantly decreased the frequency of mPSCs (*P < 0.05 and **P < 0.01). Bottom: column plots summarize the average values of AMPA-glutamate (left; mEPSCs) and GABA_A_ (right, mIPSCs) receptor mediated miniature events, pharmacologically isolated. In s-GO treatments (50 μg/mL) a significant decrease in the frequency of both types of miniature was detected (*P < 0.05 and **P < 0.01, respectively).

mPSCs (Figure 2) were recorded in a subset of control (*n* = 12) and s-GO treated (*n* = 13) neurons by application of TTX (1 μM), to block voltage-gated sodium channels. As this treatment impairs the generation of action potentials, mPSCs reflect the stochastic release of vesicles from the presynaptic terminals at individual synapses impinging onto the recorded neuron. Their frequency depends on the pre-synaptic release probability and on the number of synaptic contacts, while their amplitude depends on postsynaptic receptor sensitivity (Raastad et al., 1992). In neurons exposed to low (10 μg/mL) s-GO, mPSCs frequency was not affected (from 19 ± 3 Hz in control to 15 ± 3 Hz in s-GO treated slices; plot in Figure 2). When investigating the impact of higher graphene doses (25 and 50 μg/mL), we detected a significant difference (*P* < 0.05 and *P* < 0.01, respectively; two-way ANOVA) in mPSCs frequency (from 20 ± 3 Hz to 13 ± 2 Hz in s-GO 25 μg/mL and from 16 ± 3 Hz to 6 ± 1 Hz in s-GO 50 μg/mL). s-GO did not affect the amplitude of the recorded events (from 27 ± 6 pA in controls to 32 ± 5 pA in s-GO 10 μg/mL; from 29 ± 5 pA in controls to 27 ± 4 pA in s-GO 25 μg/mL; from 33 ± 8 pA in controls to 30 ± 6 pA in s-GO 50 μg/mL).

In s-GO neurons (50 μg/mL) we pharmacologically (see “Materials and Methods” section) isolated AMPA-receptor (AMPAR) mediated glutamatergic mEPSCs (*n* = 13) or GABA_A_-receptor mediated mIPSCs (*n* = 13), both detected as inward currents in our recording conditions (Medelin et al., 2016). mEPSCs and mIPSCs frequency values were similarly reduced by s-GO when compared to control slices (for mEPSPs in controls 14 ± 4 Hz, *n* = 12; in s-GO 7 ± 2 Hz, *n* = 13; *P* < 0.05, Student’s *t*-test; histograms in Figure 2, bottom-left panel; for mIPSCs in controls 13 ± 4 Hz, *n* = 12; in s-GO 5 ± 2 Hz, *n* = 13; *P* < 0.05, Student’s *t*-test; histograms in Figure 2, bottom-right panel).

### s-GO Exposure at High Doses Induced Microglial Proliferation

To investigate tissue reactivity accompanying s-GO ability to alter synaptic signaling, we used the highest dose tested, namely the s-GO at 50 μg/mL. In organotypic slice cultures, neuroglia resident cells are mainly represented by astrocytes (GFAP positive cells) and microglia (Iba1 positive cells; Medelin et al., 2018).

**Figure 3:**
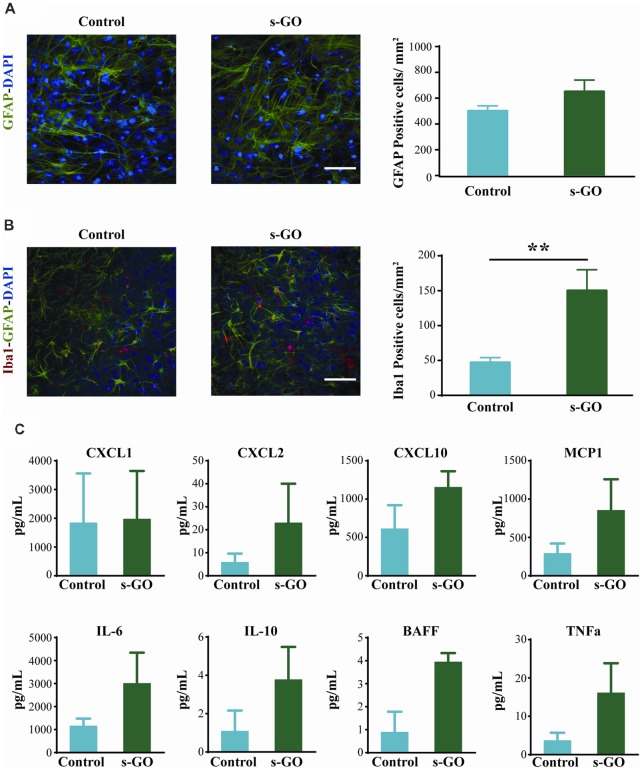
Tissue reactivity in organotypic spinal cultures exposed to s-GO at high dose. In **(A)**, GFAP immune-labeling to visualize astrocytes in Control and s-GO treated slices (50 μg/mL). Both cultures were labeled for GFAP (in green) and nuclei (DAPI; in blue) Scale bar 100 μm. Note that the GFAP+ cell density did not differ between the two conditions (column plot). In **(B)**, immunofluorescence images are shown to visualize glial and microglial cells in Control and s-GO (50 μg/mL) treated slices (anti-Iba1, in red; anti-GFAP in green; DAPI in blue). Scale bar 50 μm. Note that Iba1^+^ cell density was significantly (**P < 0.01) increased by s-GO (50 μg/mL; right column plot). **(C)** Plots summarize the Milliplex assay measures to assess the production of the following cytokines in organotypic culture supernatant: IL6, IL10, TNFα and BAFF and chemokines: CXCL1, CXCL2, CXCL10 and MCP1 in the presence or absence of s-GO, after 2 weeks *in vitro*. Column graphs report mean values ± SEM of five independent experiments.

GFAP-positive astrocytes are not immune cells *per se*, but can, under certain conditions, contribute to the immune response (Farina et al., 2007). In organotypic cultures upon 2 weeks of culturing, these cells are usually characterized by a stellate-like morphology (Figure 3A; Avossa et al., 2003) and their density was not significantly altered by s-GO treatment (Figure 3A, right histograms; 500 ± 70 GFAP-positive cells/mm^2^ in control and 650 ± 90 GFAP-positive cells in s-GO; *n* = 13 visual fields each). Iba1-positive microglia cells are known mediators of CNS inflammation. In contrast to astrocytes, the density of Iba1-positive cells was significantly (*P* < 0.01, Student’s *t*-test) increased in slices exposed to s-GO (47 ± 17 Iba1-positive cells/mm^2^, *n* = 10 fields in control and 150 ± 30 Iba1-positive cells/mm^2^, *n* = 11 fields for s-GO; Figure 3B).

To further investigate glia cell reactivity to s-GO treatment in complex systems, we measured from the spinal cord cultures supernatant (*n* = 6 slices for each conditions) the presence of cytokine and chemokine after 2 weeks of continuous exposure. In s-GO we detected an increased trend of expression, when compared to controls, of CXCL2, and MCP1, T lymphocytes and monocytes recall factors, and IL6, IL10, BAFF and TNFα, cytokines responsible for pro-inflammatory responses (IL6, TNFα), regulatory function (IL10) and homeostatic B cell survival (BAFF), however the profiles of soluble factors production obtained from our analysis, did not reach statistical significance (Figure 3C). These observations are in line with a limited or even absent sustained activation of microglial cells toward both polarized forms: M1 and M2, or most probably toward an intermediate one (Kabba et al., 2018).

### s-GO Exposure Induces Microglial Proliferation in Neuroglial Cultures

**Figure 4:**
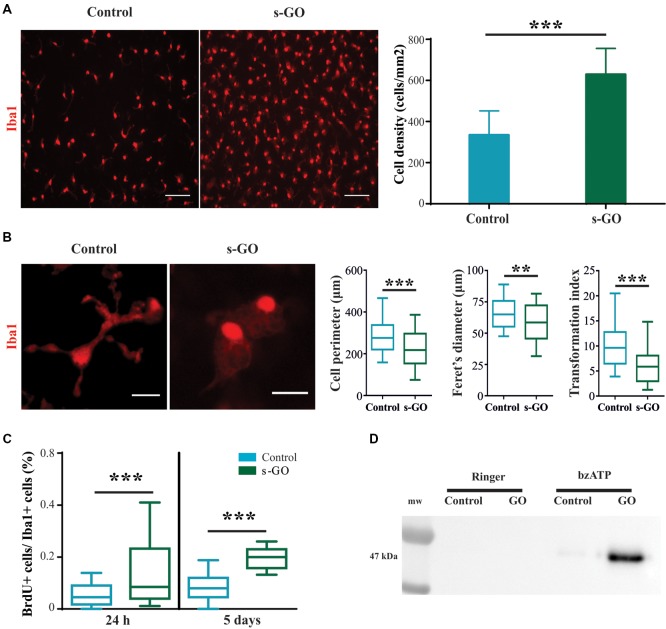
Increased cell reactivity in pure microglia cell cultures exposed to s-GO. In **(A)**, immunofluorescence micrographs visualize microglia by Iba1 labeling (in red) in Control and s-GO treated cultures (10 μg/mL). Scale bar 50 μm. The column graph summarizes microglial density in the two conditions. Note that Iba1^+^ cell density is significantly (***P < 0.001) higher in cultures treated by s-GO. In **(B)**, high magnification confocal micrographs of Iba1^+^ cells highlight their morphology in the two conditions. The right column plots summarizes perimeter, Ferret’s maximum diameter and transformation index, parameters that describe cellular ramification. All of them are significantly reduced after s-GO exposure (***P_perimeter_ < 0.0001, **P_Feret’s diameter_ < 0.001, ***P_transformation index_ < 0.0001; Mann-Whitney test). In **(C)**, box plots show the bromodeoxyuridine (brdU^+^)/Iba1^+^ ratio measured in isolated microglial cultures 24 h and 5 days after s-GO exposure (10 μg/mL; ***P < 0.001). In **(D)**, western blot analysis of the Microvesicles (MVs) marker flotillin-1 in each condition. MVs were isolated from glial cultures incubated with s-GO (10 μg/mL) for 6 days and then treated or not with benzoyl-ATP (bzATP).

The increased density of microglial cell in the absence of significant increased production and release of chemokines and cytokines after 2 weeks exposure to high s-GO dose, prompted us to directly investigate the effects of s-GO on microglial cell types in isolated glial preparations, obtained from early post-natal rats (P2-P3). Due to the relatively low-cell density typical of cultures comprising isolated Iba1-positive cells, as shown in Figure 4A (control), we exposed the cells for 5 days to a lower (10 μg/mL) dose of s-GO. s-GO readily increased Iba1-positive cell-density, as shown in Figure 4A, a response reminiscent of the one observed in organotypic slices (Figure 3B). In order to assess whether s-GO sheets induced microglia reactivity, we analyzed the cellular shape, a traditionally accepted index of the phenotypes microglia acquires when entrained in tissue responses. In particular, a highly ramified shape is linked to a surveillant state in which microglia actively monitors the surrounding environment. On the other hand, an ameboid phenotype may indicate the transition to the activated, pro-inflammatory state (Saijo and Glass, 2011). Consistent with a transformation from a ramified to an ameboid phenotype, the perimeter, Feret’s maximum diameter and transformation index (plot in Figure 4B), significantly decreased (*P*_perimeter_ < 0.0001, *P*_Feret’s diameter_ = 0.0035, *P*_transformation index_ < 0.0001; Mann-Whitney test) after 6 days of s-GO exposure, compared to control (*n* = 10 visual fields for both conditions; three different cultures series). The measured parameters reliably describe morphological changes and cell length. In particular, the Feret’s maximum diameter is defined as the highest distance between any two points along the cell perimeter while transformation index describes cellular ramification.

Next, we analyzed the number of cycling cells present in each culture group (control and s-GO). For this purpose, cells were pulsed at 24 h and at 5 days with 10 mM BrdU prior to fixation. The number of cells that had incorporated the nucleotide analog was then assessed by immunofluorescence, using anti-BrdU-specific antibodies. In Figure 4C the box plot shows the BrdU^+^/Iba1^+^ ratio, an index of microglial cells that incorporated BrdU in the newly synthesized DNA during cell division, providing a quantitative measure of proliferative capacity of cells (Nowakowski et al., 1989). The higher BrdU^+^/Iba1^+^ ratio was already significant at 24 h of s-GO exposure (median_GO_ = 0.85; median_control_ = 0.08), suggesting an early interaction of microglia with small graphene sheets. The increased proliferation was more pronounced after 5 days of incubation with s-GO (median_GO_ = 0.2; median_control_ = 0.45; *P* < 0.001, Mann-Whitney test, for both time points; *n* = 10 fields for each condition; four different cultures).

MVs, released from almost all cell brain types, are, in general, an emerging intercellular communication over long-range distance. In particular, MVs discharged by microglial cells represent a secretory pathway for inflammatory cytokine (Turola et al., 2012) potentially promoting propagation of neuroinflammatory responses in the brain. We measured the release of MVs from isolated microglial cultures in control or exposed to s-GO by western blot analysis for the protein flotillin-1, a marker of lipid rafts that are specific plasma membrane regions were the probability of MVs release is higher (Figure 4D; Rauti et al., 2016). Pharmacological stimulation by bzATP induces only a slight release of MVs in isolated microglial cells, as depicted by the particularly weak signal in the specific band (Figure 4D). Interestingly, bzATP stimulation in the presence of s-GO triggers a massive microglia shedding of MVs, shown by the high intensity of the band (Figure 4D).

### Localization of s-GO in Microglial Cells

**Figure 5:**
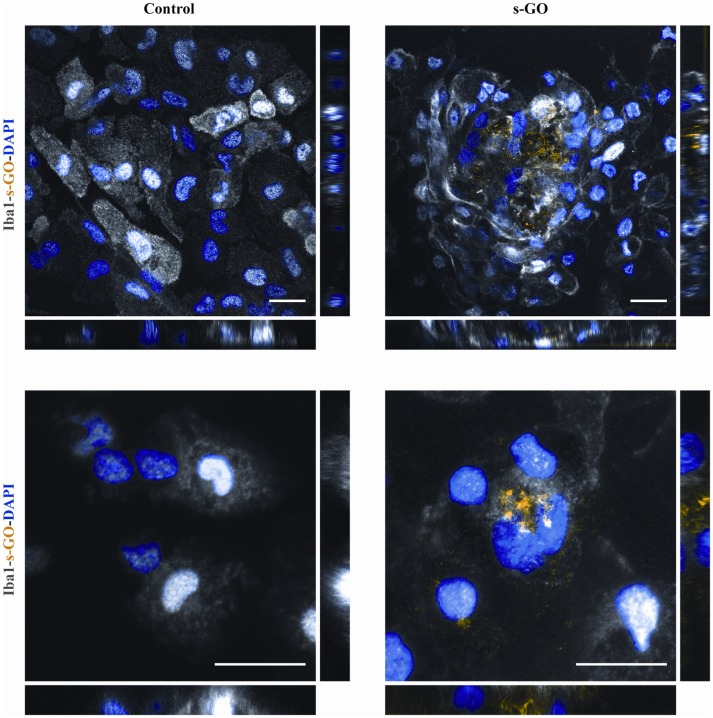
s-GO activates microglia phagocytosis in pure neuroglia cultures. Representative confocal reconstruction of microglial cells in Control or s-GO (10 μg/mL) treated cultures. Scale bar 20 μm. In the bottom panel, for both conditions a 60× zoom (50 × 50 μm field) of a single microglial cell is shown. Scale bar 20 μm. Cultures are immunostained for Iba1 (in gray), and DAPI (in blue), s-GO is visualized by the reflection mode of the confocal system (in yellow). s-GO sheets are visible as aggregates colored in yellow inside the cell.

We then directly investigated the fate of s-GO in isolated microglia cultures using confocal microscopy reconstructions. Iba1-positive cells (two different culture series) were exposed for 3 days to s-GO (10 μg/mL). We tested the presence of s-GO sheets within Iba1 positive cells by operating the confocal microscopy under reflection mode, which allows the visualization of s-GO (Jung et al., 2007; Kim et al., 2010; Bramini et al., 2016; Chiacchiaretta et al., 2018). Figure 5 shows confocal reconstructions of control and treated Iba1 positive cells. In s-GO-treated cells, GO nano-sheet aggregates (in yellow, reflection mode) were detected inside microglial cells (in gray, Iba1^+^) by z-stack reconstruction (Figure 5, top panels: 40×; 100 × 100 μm^2^ visualized area). In Figure 5, high magnification confocal micrographs (control and s-GO treated, bottom panels) are shown (60×; 50 × 50 μm^2^ visualized area) depicting a single microglia (gray) cell co-localization with the reflected signal of graphene (yellow). The orthogonal view of the z-projection shows the XZ and YZ planes (bottom side and right side of the z-stack reconstructed image, respectively) of the acquired fields. As expected from cells that act as macrophages, the material was internalized and stocked inside the cell, forming small aggregates, appreciable by the orthogonal reconstruction. The signal of s-GO was not present in control cells, either in the z-stack reconstruction or in the orthogonal planes (Figure 5, left panels).

## Discussion

We used here organotypic spinal cord cultures to test tissue responses to s-GO prolonged exposure. In particular, we were interested in assessing microglia reactivity in cultured neural explants, where immune resident cells are present, but not supported by the peripheral ones.

Organotypic spinal slices represent a biological model of segmental spinal microcircuit useful for studying the dynamics of intrasegmental processes. A long series of studies has indicated that the organotypic cultures from mouse spinal cord represent a valuable *in vitro* model system to study the mechanisms of development, neurogenesis, glial differentiation, myelination, muscle formation, and synaptogenesis leading to early post natal features, upon 2 weeks of culturing (Avossa et al., 2003; Rosato-Siri et al., 2004; Fabbro et al., 2007; Furlan et al., 2007; Sibilla et al., 2009; Medelin et al., 2016). More recently, we confirmed the presence of heterogeneous neuroglial cells after 2 weeks of *in vitro* growth and we further documented in organotypic spinal tissue the induction, by short-term incubation with pro-inflammatory CKs cocktail, of a reliable release of cytokines and chemokines, mostly due to the local generation and delivery of inflammatory factors (Medelin et al., 2018). Thus, our experimental model is ideally suited to dissect spinal resident cells ability in modulating local inflammatory tissue reactivity.

The major result of the present investigation is that the long-term accumulation of s-GO (when delivered at high doses) affected resident microglia and, in the absence of an effective clearance, may induce a subtle, although chronic, reactive state, potentially trimming down synaptic activity, as physiologically occurring during development (Paolicelli et al., 2011). In fact, in our experiments, both GABAR- and AMPAR-mediated mPSCs were reduced in frequency upon s-GO exposure at high concentrations. The reduction in miniatures’ frequency, but not in their amplitude, strongly suggests a reduction in the number of synapses or of release sites (Rauti et al., 2016). This down-regulation of synapses is apparently not due to a general cell membrane disruption or to neuronal apoptosis. In fact, we never detected alterations in basic electrophysiological parameters, reflecting neuronal health and membrane integrity (Carp, 1992; Djuric et al., 2015). This indicated, together with the absence of up-regulated apoptosis that the synaptic events diminished not as a consequence of direct neuronal damage brought about by s-GO.

In organotypic slices, both glutamate- and GABA-mediated synapses were down-regulated by s-GO, this result differs from our previous report, where s-GO down-regulated selectively glutamatergic release sites in hippocampal cultures (Rauti et al., 2016). Considering the similarity of the s-GO batch used here compared to our previous study (Supplementary Figure S1), the lack of specific synaptic targeting may be related to several factors, such as the diverse CNS regions tested, i.e., ventral spinal cord vs. hippocampus, the initially more immature stage of network and synapse development (embryonic vs. postnatal) or the s-GO higher concentrations. In this context, it is relevant to note the s-GO delivery modality used here: s-GO was accumulated in the fibrin glue embedding the spinal culture and, presumably, was from here released along 2 weeks of culturing. Thus, the potential formation of a protein corona might have affected the nanoparticle biological fate (Hadjidemetriou et al., 2015) favoring active phagocytosis by neuroglia and preventing the direct synapse interference described in our previous report (Rauti et al., 2016). It is tempting to speculate that the presence in the spinal explants of resident neuroglia resulted in active phagocytosis restricted to intrinsic microglia, without the involvement of blood cells such as macrophages. Such an activation could have induced a generic microglia response, known as synaptic stripping, ultimately leading to indiscriminate synapse reduction (Kettenmann et al., 2013).

In spinal organotypic slices exposed to s-GO, the presence of tissue reactivity is indicated only by the observed increase in microglia cell-density, in the absence of increased GFAP+ astrocytes, suggesting only a mild state of reactivity (Olson, 2010; Liddelow et al., 2017; Okada et al., 2018). Interestingly, the cytokines and chemokines profiles measured in organotypic cultures supernatant, although slightly altered, were not significantly increased after 2 weeks of s-GO exposure. In this regard, we did not find an increase in IL-6, that is produced by astrocyte following, for example, a proinflammatory stimulation (Ulivieri et al., 2016). Therefore, in this model, we may hypothesize that microglial proliferative response with no significant variation in cytokines production after 2 weeks, does not involve a shift into the M1 phenotype.

The results in this study obtained with isolated Iba1^+^ cells are in support of a direct activation of microglial cells as a consequence of active s-GO phagocytosis. s-GO boosted microglia proliferation leading to a significantly higher cell density in pure neuroglia cultures, accompanied by the typical morphological switch from a ramified to an ameboid phenotype (Nimmerjahn et al., 2005; Kettenmann et al., 2011; Saijo and Glass, 2011; Cherry et al., 2014), suggestive of an active role of Iba1-positive cells in the tissue reaction to graphene, even in the virtual absence of other cell types. The direct activation of microglia was apparently related to fast internalization of s-GO flake aggregates that occurs during the first 24 h after the exposure.

We thus suggest that s-GO, accumulated *via* the fibrin glue, activates resident microglia phagocytosis. This hypothesis is also supported by the observation that pure microglial cultures grown and exposed to s-GO in a serum-free medium did not show a proliferation boost comparable to that found in the presence of FBS (Supplementary Figure S3). As already discussed above, the interaction of nanoparticles such as s-GO with biological fluids may affect their fate and effectiveness. In fact, the corona formation takes place not only in the presence of plasma proteins like fibrinogen but also with proteins present in the serum (Gräfe et al., 2016). Therefore, the proliferative response found in both pure microglial cultures and organotypic slices exposed to s-GO, despite their different origin and architecture, may be explained by an increase of the efficacy in the uptake of the material (Walkey et al., 2012) by microglia and mediated by s-GO interactions with the proteins pool of plasma and serum.

An additional indication of microglia reactivity in s-GO was provided by the increased release of shed vesicles induced by bzATP. MVs released by microglia have been reported to affect synaptic activity, mainly acting at the presynaptic site of the excitatory synapses, but increasing synaptic activity and glutamate release in primary cultures (Antonucci et al., 2012) thus it seems unfeasible that the down-regulation in synaptic activity detected in this study be mediated by MVs release.

One possibility is that, in the current experimental conditions, the global reduction in synaptic activity is mostly due to the emergence of chronic, although mild, tissue reactivity. Such reactivity follows microglia phagocytic activity of protein-decorated s-GO flakes. Ultimately, we have to ask ourselves whether these data indicate a potential* in vivo* inflammatory response due to s-GO sheets. There are several relevant aspects that limit reporting it as an inflammatory response. First of all, the conditions we tested involved the delivering of s-GO to a “closed” biological system, allowing only resident macrophages to interact with it and limiting the contribution of neighbor tissues in the interaction with this material, as it would happen in an* in vivo* model. The lack of significance in the chemokine and cytokine increase in concentration suggests a mild immune response that may not necessarily lead to a pathological inflammatory state or alternatively indicates a return to a physiological condition, from an intermediate immune activation. Last, astrocytes, which depend on microglia activation to be polarized to pro-inflammatory cells did not show, in this context, reactive gliosis. To note, a recent report (Song et al., 2014) investigated microglia pro- and/or anti-inflammatory responses when challenged by different graphene structures, and documented anti-inflammatory effects of 3D-graphene foams.

Microglia produces immune mediators secondary to neuronal stimulation, (i.e., tissue injury), or following a direct stimulus to microglia itself. Here, we report an increased proliferation rate of Iba1-positive cells, suggestive of microglia activation, and consistent with morphological observations. However, the detected cell activation seems to involve a group of cells, not all of them, and this may explain the fact that we observed only a trend toward increased production of immune factors, without significant variations. We may hypothesize that the fraction of activated microglia includes cells in direct contact to s-GO. Indeed, microglia is often reported to function similarly to other myeloid cells, the macrophages, able to scavenge the environment, perform phagocytosis, antigen presentation and to react to contact with nano materials (Aldinucci et al., 2013; Jin and Yamashita, 2016) in order to maintain CNS homeostasis, with both detrimental and beneficial effects. It is important to underline that eventually the pro-inflammatory molecules, present before and after s-GO contact, were not inducing astrogliosis. Therefore, as a side effect, our* in vitro* model allows the direct observation and study of material/neuron interactions in the presence of glial cells, and also simulates accumulation of material in CNS providing useful insights on the potential consequences.

The function of microglia, brain macrophages, is still poorly understood. In mice there are at least two subtypes: inflammatory and non-classical patrolling cells (Nimmerjahn et al., 2005), and also a CNS region-dependent microglial heterogeneity has been suggested, suggesting that microglia, although in many different ways, is constantly activated. In this complex picture, we were interested in investigating whether s-GO induced an inflammatory reaction. Our results indicate that this was not the case. Further studies should indeed address the interplay between microglia and s-GO, for example by single cell RNA sequencing (Matcovitch-Natan et al., 2016), a method that has sufficient sensibility to determine the functional/phenotypic response of these important brain-macrophage cell population.

## Data Availability

The datasets generated for this study are available on request to the corresponding author.

## Author Contributions

MM and RR performed all cell biology, electrophysiology, and confocal experiments and analysis. AR and KK contributed to the synthesis and characterization of thin graphene oxide (s-GO). EB and CB designed and performed the supernatant measures. CB and LB conceived the study and the experimental design. CB, RR and LB wrote the manuscript.

## Conflict of Interest Statement

The authors declare that the research was conducted in the absence of any commercial or financial relationships that could be construed as a potential conflict of interest.

## Supplementary Material

FIGURE S1 Physicochemical characterization of s-GO. **(A)** AFM height image of s-GO used in the present study. **(B)** Lateral dimension and **(C)** thickness distributions of s-GO nanosheets analyzed by AFM. **(D)** Table summarizes the full characterization of the s-GO batch used here and compares with the s-GO used in our previous study. The similar physicochemical properties of both s-GOs reveal low batch-to-batch variation.

FIGURE S2 Confocal reconstructions, acquired via reflection mode configuration, of s-GO nanosheets dispersed in the fibrin glue at different concentrations. The image was reconstructed by stitching 3 × 3 visual fields acquired at 20× magnification. Scale bar: 200 μm.

FIGURE S3 brdU+/Iba1+ ratio measured in isolated microglial cultures 24 h after s-GO exposure (10 μg/mL) in a FBS-free medium. No significant differences were observed between the two conditions (mean ± SEM).
