# Supplementary figures and images for: 3D Organotypic Spinal Cultures: Exploring Neuron and Neuroglia Responses Upon Prolonged Exposure to Graphene Oxide
Source: Front Syst Neurosci. 2019 Jan 24;13:1. doi: 10.3389/fnsys.2019.00001 (PMC6354065; doi:10.3389/fnsys.2019.00001)

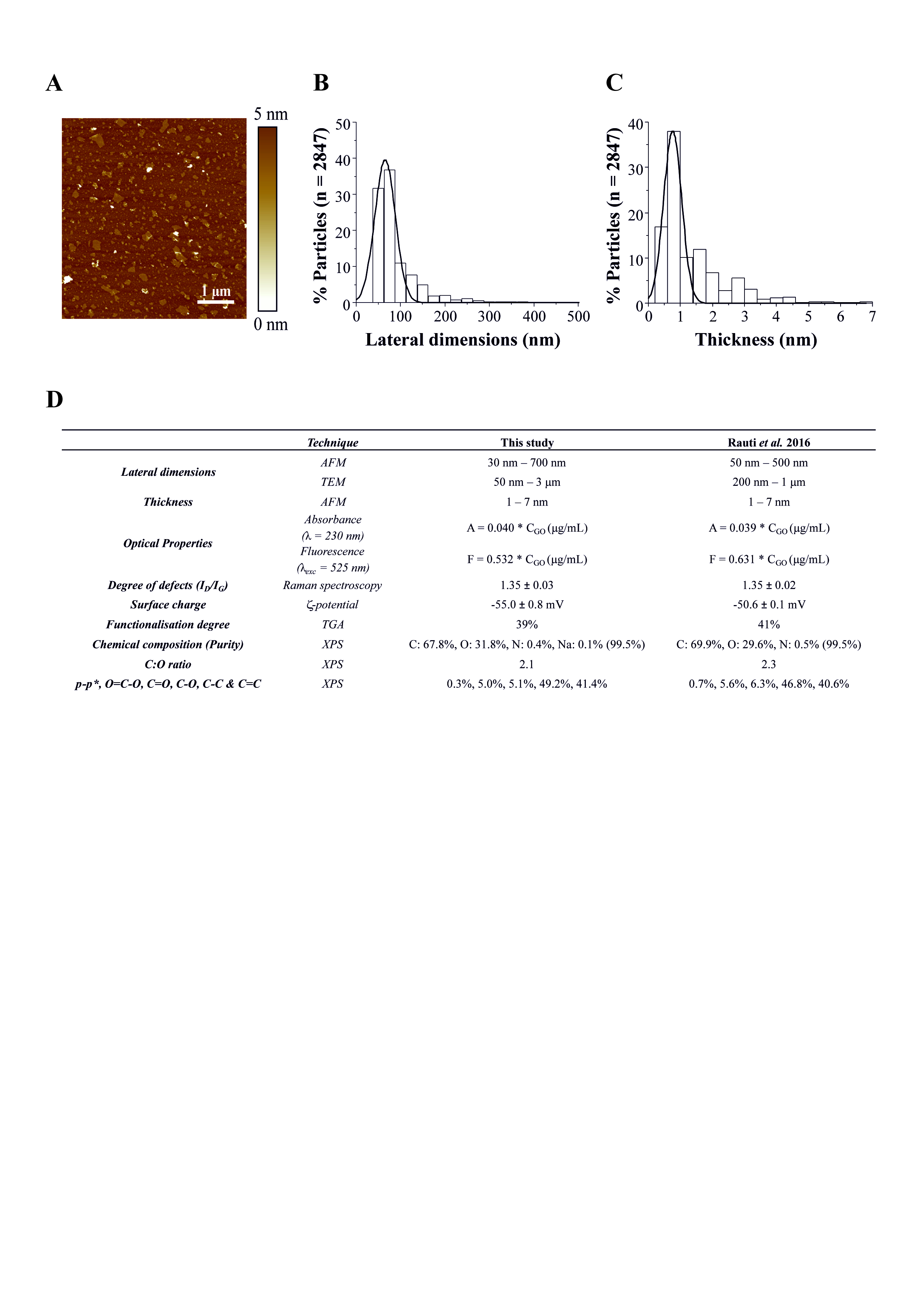

Supplement: FIGURE S1 — Physicochemical characterization of s-GO. (A) AFM height image of s-GO used in the present study. (B) Lateral dimension and (C) thickness distributions of s-GO nanosheets analyzed by AFM. (D) Table summarizes the full characterization of the s-GO batch used here and compares with the s-GO used in our previous study. The similar physicochemical properties of both s-GOs reveal low batch-to-batch variation. [file Image_1.TIF]

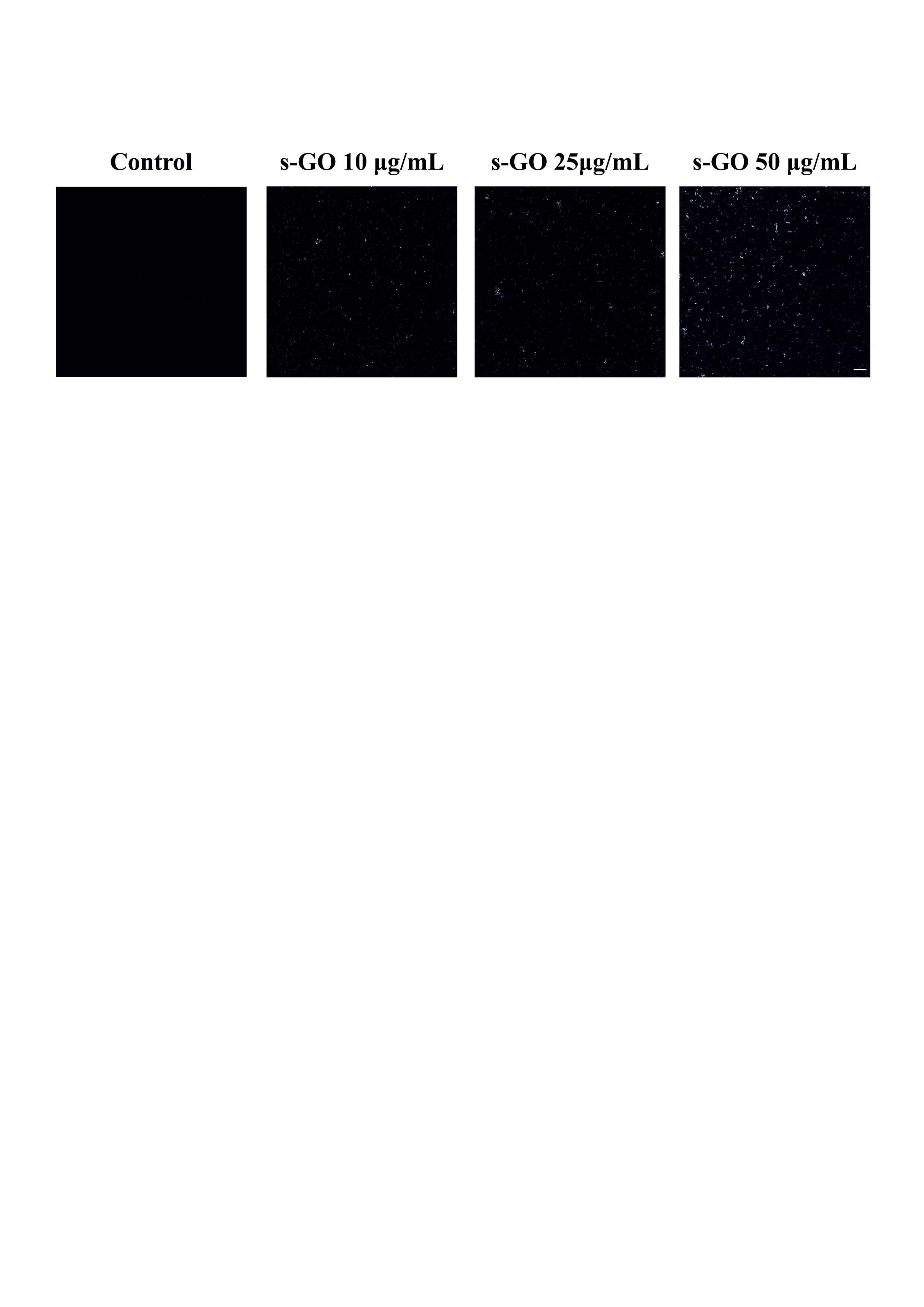

Supplement: FIGURE S2 — Confocal reconstructions, acquired via reflection mode configuration, of s-GO nanosheets dispersed in the fibrin glue at different concentrations. The image was reconstructed by stitching 3 × 3 visual fields acquired at 20× magnification. Scale bar: 200 μm. [file Image_2.TIF]

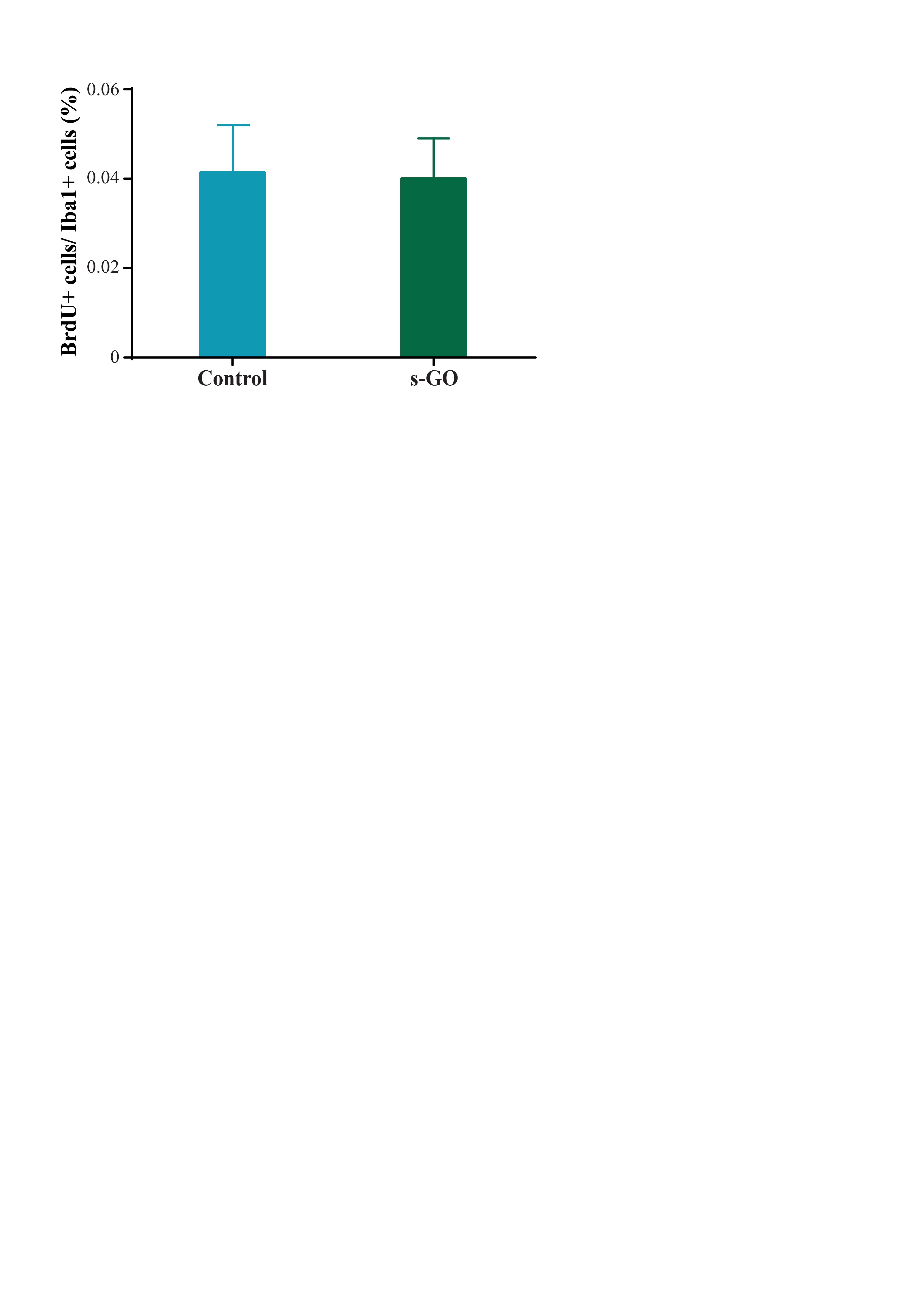

Supplement: FIGURE S3 — brdU+/Iba1+ ratio measured in isolated microglial cultures 24 h after s-GO exposure (10 μg/mL) in a FBS-free medium. No significant differences were observed between the two conditions (mean ± SEM). [file Image_3.TIF]
